# Supplementary material for: COZOID: contact zone identifier for visual analysis of protein-protein interactions
Source: BMC Bioinformatics. 2018 Apr 6;19:125. doi: 10.1186/s12859-018-2113-6 (PMC5889581; doi:10.1186/s12859-018-2113-6)
Supplement: Supplementary file 2 — Software build. Executable binary file of the software tool. (ZIP 118,784 kb) [file 12859_2018_2113_MOESM2_ESM.zip › cozoid/platform/docs/testng-6.5.1-javadoc.zip!/org/testng/IHookable.html]

IHookable (TestNG)


---


|  |  |  |
| --- | --- | --- |
| **Overview** \| **Package** \| **Class** \| **Tree** \| **Deprecated** \| **Index** \| **Help** | |  |
| **PREV CLASS**   **NEXT CLASS** | **FRAMES**    **NO FRAMES**     **All Classes** |  |
| SUMMARY: NESTED \| FIELD \| CONSTR \| METHOD | DETAIL: FIELD \| CONSTR \| METHOD |  |


---


## org.testng Interface IHookable

**All Superinterfaces:**: ITestNGListener

---

``` public interface IHookable extends ITestNGListener ```

If a test class implements this interface, its run() method
will be invoked instead of each @Test method found. The invocation of
the test method will then be performed upon invocation of the callBack()
method of the IHookCallBack parameter.
This is useful to test classes that require JAAS authentication, which can
be implemented as follows:

```
 public void run(final IHookCallBack icb, ITestResult testResult) {
   // Preferably initialized in a @Configuration method
   mySubject = authenticateWithJAAs();

   Subject.doAs(mySubject, new PrivilegedExceptionAction() {
     public Object run() {
       icb.callback(testResult);
     }
   };
 }
```

**Author:**
:   cbeust
    Jan 28, 2006

---

| **Method Summary** | |
| --- | --- |
| `void` | `run(IHookCallBack callBack, ITestResult testResult)` |

**Method Detail**

### run

```
void run(IHookCallBack callBack,
         ITestResult testResult)
```


---


|  |  |  |
| --- | --- | --- |
| **Overview** \| **Package** \| **Class** \| **Tree** \| **Deprecated** \| **Index** \| **Help** | |  |
| **PREV CLASS**   **NEXT CLASS** | **FRAMES**    **NO FRAMES**     **All Classes** |  |
| SUMMARY: NESTED \| FIELD \| CONSTR \| METHOD | DETAIL: FIELD \| CONSTR \| METHOD |  |


---
